# RETRACTION: miR‐375 Inhibits the Proliferation and Invasion of Nasopharyngeal Carcinoma Cells by Suppressing PDK1

**DOI:** 10.1155/bmri/9836269

**Published:** 2026-06-27

**Authors:** BioMed Research International

RETRACTION: X. Jia‐yuan, S. Wei, L. Fang‐fang, D. Zhi‐jian, C. Long‐he, L. Sen, “miR‐375 Inhibits the Proliferation and Invasion of Nasopharyngeal Carcinoma Cells by Suppressing PDK1,’” BioMed Research International 2020, no. 1 (2020): 9704245, 10.1155/2020/9704245

The above article, published online on 23 March 2020 in Wiley Online Library (http://wileyonlinelibrary.com), and its correction (https://onlinelibrary.wiley.com/doi/10.1155/2020/3595402), have been retracted by agreement between the authors, the handling Editor, Andrei Surguchov, and John Wiley & Sons Ltd. The retraction has been agreed due to errors in the Figures, specifically:

• Unexpected repeated elements between the CNE‐1/miR‐375 mimics panel and the CNE‐2/miR375 inhibitor panel in Figure 2c.
• The *β*‐Actin bands in Figure 4d appear similar to the GAPDH bands in Figure 4a of https://pubmed.ncbi.nlm.nih.gov/30237545/

The authors requested the retraction, and after assessment by the editorial board the article is retracted.

